# GABPA predicts prognosis and inhibits metastasis of hepatocellular carcinoma

**DOI:** 10.1186/s12885-017-3373-7

**Published:** 2017-05-26

**Authors:** Sheng Zhang, Kang Zhang, Piyou Ji, Xuqing Zheng, Jianbin Jin, Min Feng, Pingguo Liu

**Affiliations:** 1 Department of Hepatobiliary Surgery, ZhongShan Hospital Xiamen University, Xiamen, China; 2 Fujian Provincial Key Laboratory of Chronic Liver Disease and Hepatocellular Carcinoma (Xiamen University Affiliated ZhongShan Hospital), Xiamen, China

**Keywords:** Hepatocellular carcinoma, GABPA, Prognosis, Metastasis, E-cadherin

## Abstract

**Background:**

Increasing evidence indicates that abnormal expression of GABPA is associated with tumor development and progression. However, the function and clinicopathological significance of GABPA in hepatocellular carcinoma (HCC) remain obscure.

**Methods:**

The mRNA and protein expression of GABPA in HCC clinical specimens and cell lines was examined by real-time PCR and western blotting, respectively. Follow-up data were used to uncover the relationship between GABPA expression and the prognosis of HCC patients. HCC cell lines stably overexpressing or silencing GABPA were established to explore the function of GABPA in HCC cell migration and invasion by Transwell and wound healing assays in vitro and in a xenograft model in vivo. Restoration of function analysis was used to examine the underlying molecular mechanisms.

**Results:**

GABPA was downregulated at the protein and mRNA levels in HCC tissues compared with adjacent normal tissues. Decreased GABPA expression was correlated with alpha-fetoprotein levels (*P* = 0.001), tumor grade (*P* = 0.017), and distant metastasis (*P* = 0.021). Kaplan-Meier survival analysis showed that patients with lower GABPA expression had significantly shorter survival times than those with higher GABPA (*P* = 0.031). In vivo and in vitro assays demonstrated that GABPA negatively regulated HCC cell migration and invasion, and the effect of GABPA on HCC cell migration was mediated at least partly by the regulation of E-cadherin.

**Conclusions:**

Collectively, our data indicate that GABPA inhibits HCC cell migration by modulating E-cadherin and could serve as a novel biomarker for HCC prognosis. GABPA may act as a tumor suppressor during HCC progression and metastasis, and is a potential therapeutic target in HCC.

**Electronic supplementary material:**

The online version of this article (doi:10.1186/s12885-017-3373-7) contains supplementary material, which is available to authorized users.

## Background

Hepatocellular carcinoma (HCC), which accounts for 85–90% of primary liver cancers, is a common malignancy worldwide and the second leading cause of cancer-related mortality [1]. Especially in China, where it is accompanied by a high infection rate of hepatitis B virus, the importance of this disease should not be underestimated [2]. Advances in modern medicine have resulted in the development of techniques for the diagnosis and therapy of HCC [3–5]. Surgical resection and liver transplantation remain the treatment of choice for HCC patients in the early stage; however, most patients are at an advanced stage at presentation. Despite the fact that research into the treatment of HCC has been ongoing for decades, the prognosis and survival of HCC patients remain disappointing because of recurrence and metastasis [6, 7]. Moreover, the mechanism underlying HCC development remains unclear, although many molecular biomarkers involved in HCC have been identified. Therefore, elucidating the potential mechanisms underlying HCC occurrence and development is critical to identify effective treatments for this disease.

GA binding protein (GABP) transcription factor alpha subunit (GABPA) is a subunit of the obligate heteromeric E twenty-six (ETS) transcription factor GABP. It harbors a highly conserved ETS motif that acts as a DNA-binding motif [8, 9], as well as a protein-protein interaction domain for binding to the GABP beta subunit [10]. GABPA regulates a broad range of genes involved in embryonic development, innate and acquired immunity, myeloid and hematopoietic stem cell differentiation, cell cycle progression, and migratory properties, and plays a role in certain human diseases.

GABPA regulates the expression of genes involved in mitochondrial function, and its inactivation results in early embryonic lethality [11]. In addition, GABPA conditional deletion in mouse embryonic fibroblasts markedly decreases Tfb1m expression and reduces mitochondrial mass and protein synthesis, ATP production, and oxygen consumption [12]. Deficiency of GABPA leads to a profound defect in B cell development and a compromised humoral immune response, in addition to thymic developmental defects [13].

GABPA is involved in the maintenance and differentiation of hematopoietic stem and progenitor cells by activating the transcription of DNA methyltransferases and histone acetylases [14]. In addition, GABPA is required for myeloid differentiation through the activation of the integrin alpha M promoter [15]. Yang et al. reported that GABPA is required for myeloid differentiation in part by regulating the transcriptional repressor Gfi-1 [16].

GABPA plays a major direct role in cell cycle progression. Conditional deletion of GABPA in mouse embryonic fibroblasts (MEFs) causes G1/S cell cycle arrest [17], and reduces the numbers of cells entering the cell cycle [18]. GABPA regulates cell survival and cell cycle progression through Yes-associated protein [19]. GABPA is activated in a cell cycle-dependent manner and regulates the expression of genes related to cell cycle progression [20]. Perdomo-Sabogal et al. used chromatin immunoprecipitation (ChIP) and comparative genomic approaches to identify newly evolved GABPA binding sites in 17 genes associated with a series of human diseases [21]. Furthermore, a previous study showed that GABPA plays an important role in human chronic myelogenous leukemia (CML) and affects imatinib sensitivity [22]. GABPA is required for the entry of hematopoietic stem cells into the cell cycle through the regulation of PRKD2 [23].

A previous study showed that ablation of GABPA weakens the migratory properties of vascular smooth muscle cells by modulating the expression of kinase interacting with stathmin (KIS), which affects the phosphorylation and activity of p27 [18]. Odrowaz and Sharrocks confirmed that GABPA plays a complex role in controlling breast epithelial cell migration by directly affecting the expression of RAC2 and KIF20A [24]. However, studies on the role of GABPA in human cancer are rare, and whether GABPA is involved in HCC cell invasion and migration remains unclear.

The loss of E-cadherin, a calcium-dependent cell-cell adhesion protein, is associated with tumor migration, invasion, and poor prognosis. Epithelial cells can acquire a fibroblastoid morphotype accompanied by the acquisition of invasive and metastatic abilities in response to E-cadherin downregulation. Several transcription factors including Snail, Slug, and Twist among others are involved in the repression of E-cadherin gene transcription and the induction of epithelial-mesenchymal transition (EMT). However, to the best of our knowledge, there are no studies addressing the relationship between GABPA and E-cadherin expression.

In the present study, stably overexpressing and silencing GABPA cell lines were established to examine the potential role of GABPA in the regulation of HCC cell migration and invasion. GABPA expression was detected in human paired HCC tissue samples by western blotting and real-time PCR, and GABPA function was tested in vitro and in vivo. Finally, we investigated the potential molecular mechanisms underlying the effect of GABPA on HCC cell migration.

## Methods

### Cell culture

Six common HCC cell lines, MHCC-97H, PLC, BEL-7402, SMMC-7721, Huh7, SK-Hep1, and LO2, a normal liver cell lines, were purchased from the cell bank of Shanghai Institute of Cell Biology (Shanghai, China). All cells were cultured in RPMI-1640 or DMEM (Invitrogen) mediums. All the mediums were added with 10% fetal bovine serum (FBS) (Hyclon) and 100 units/ml of penicillin and streptomycin (Sigma). Cell lines were cultured according to the manufacturer’s protocol. All the cell lines were grown at 37 °C, in a 5% CO_2_ atmosphere, and passaged every 2–4 days.

### Clinical samples

All of the clinical samples were obtained from chronic liver disease biological sample bank, department of Hepatobiliary Surgery, Zhongshan Hospital Xiamen University. None of the patients has received neoadjuvant therapy before surgical resection. The ethical approval was granted from the Committees for Ethical Review at the hospital. Written informed consent was also obtained from all patients based on the Declaration of Helsinki. The post-surgical patients were followed-up until September 2016.

### Lentivirus vector based shRNA and overexpression

The pSIREN-RetroQ-puro RNA interference vector, which contained an RNA interference sequence that targeted GABPA or E-cadherin, was constructed similarly to the previous description [25]. Forward and reverse short-hairpin RNAs (ShRNAs) which targeted GABPA or E-cadherin were annealed together respectively and inserted into the downstream from the promoter, finally generating the shRNA plasmid. The shRNA sequences were shown in Table 1. For GABPA over-expression plasmid, 1365 bp genomic sequence of GABPA coding region was cloned into the backbone of PBOBI-CMV vector downstream from the CMV promoter. The above mentioned plasmids and the virus packaging plasmids pMD2.G and PAX2 were transfected using the turbofect Transfection Reagent (Thermo, Cat #R0531) according to the manufacturer’s instructions. Then the HCC cells were transfected with virus-containing supernatant fluid and polybrene (10 μg/ml). Puromycin (2 μg/ml) was used for selection. Stable transfectants were maintained in conditional mediums with puromycin (1.0 μg/ml) for further analysis.

**Table 1:** Primers sequences

	Primer name	F:5′-3′	R:5′-3’
RT-PCR	GABPA	AAGAACGCCTTGGGATACCCT	GTGAGGTCTATATCGGTCATGCT
	E-cadherin	CGACCCAACCCAAGAATCTATC	AGGTGGTCACTTGGTCTTTATTC
	β-actin	ATAGCACAGCCTGGATAGCAACGTAC	CACCTTCTACAATGAGCTGCGTGTG
shRNA	GABPA-1	CCGGTGTTATCAGTAAGAAGTTCTAGCTTCAAGAGAGCTAGAACTTCTTACTGATAATTTTTTG	AATTCAAAAAATTATCAGTAAGAAGTTCTAGCTCTCTTGAAGCTAGAACTTCTTACTGATAACA
	GABPA-2	CCGGTGATCTGGATCAATAACAACCTCTTCAAGAGAGAGGTTGTTATTGATCCAGATTTTTTTG	AATTCAAAAAAATCTGGATCAATAACAACCTCTCTCTTGAAGAGGTTGTTATTGATCCAGATCA
	E-cadherin	GATCCGCACCAAAGTCACGCTGAATTTCAAGAGAATTCAGCGTGACTTTGGTGTTTTTTACGCGTG	AATTCACGCGTAAAAAACACCAAAGTCACGCTGAATTCTCTTGAAATTCAGCGTGACTTTGGTGCG
Pbobi-cmv	GABPA	GACTCTAGAGGATCCATGTACCCATACGACGTCCCAGACTACGCTACTAAAAGAGAAGCAGAGGAGC	AATTAATTCCTCGAGTTAATTATCCTTTTCCGTTTGCAGAGAAGC
ChIP RT-PCR	E-Cadherin-P1	CAGTTGCTATGATGAGCCAAGA	GGGAAGTCAGTGTTCTCCTTTG
	E-Cadherin-P2	CTCTCATTGGCCTCAATCTCTC	GCCACTGACCAGCTCATTTA
	E-Cadherin-P3	ACCACGCCTGGCTAATTT	GATCACGAGGTCAGGAGATTG
	E-Cadherin-P4	CTCACTAACCCATGAAGCTCTAC	GCCGAGGCTGATCTCAAAT
	E-Cadherin-P5	CACCTGTACTCCCAGCTACTA	GGTCTCACTCTTTCACCCAAG

### Western blot

Cultured cells were washed twice with ice-cold phosphatebuffered saline (PBS), then solubilized in a lysis buffer containing 1 mmol/L protease inhibitor cocktail (Sigma, St Louis, MO, USA) and quantified using the Bradford method. Protein lysate was separated by 6–12% sodium dodecyl sulfate polyacrylamide gel electrophoresis and then transferred to polyvinylidene difluoride membranes (Millipore, Billerica, MA, USA). After blocking the membranes with 0.05 g/mL non-fat milk, the blots were incubated with primary antibodies directly against GABPA (1:500, 21,542–1-AP, Proteintech), β-Actin (1:1000, #3700, CST), EZH2 (1:1000, #5246, CST) and E-cadherin (1:1000, 14472S, CST) at 4 °C overnight. Thereafter, the membranes were washed and incubated for 2–3 h at room temperature with the horseradish peroxidase conjugated secondary antibody. Protein bands were visualized with an enhanced chemiluminescence Reagent (K12045-D50, Advansta, USA) and quantified by densitometry via the Image-J software. The relative protein levels were calculated by comparing to the amount of β-Actin protein. Experiments were repeated in triplicate.

### RT-PCR

Total RNA was extracted from tissues samples or cells using the Trizol reagent (Ambion, Cat 15,596–026, USA) according to the manufacturer’s instructions and then quantified at 260 nm using a NanoDrop 2000 spectrophotometer (Thermo Scientific, Waltham, MA, USA). Primers were designed and synthesized by BGI-Tech (Shenzhen, China). The sequences of the primer pairs were showed in Table 1. Total RNA (2 μg) was reverse-transcribed to complementary DNA (cDNA) using an RT kit (Promega, Madison, WI, USA). And quantitative PCR was performed in triplicate using Platinum SYBR Green qPCR Super Mix-UDG reagents (Invitrogen, Carlsbad, CA, USA) on a CFX96 Touch™ sequence detection system (Bio-Rad, Hercules, CA, USA). A dissociation procedure was performed to generate a melting curve for confirmation of amplification specificity. GAPDH was used as the endogenous control, and the comparative threshold cycle (2-ΔΔCT) equation was used to calculate the relative expression levels. All above were performed following the MIQE guidelines reported in the previous research [26].

### Wound healing assay

One day before the wound healing assay performed, HCC cells were seeded in 6-well plates. Once cellular density reached nearly 100% density, cells were scraped in a straight line with a 200 μl yellow micro-pipette tip, and photographed using phase-contrast microscopy to get the original width. Then the cells were put back into incubator. In order to assess migration distance, micrographs were taken every 12 h and quantified the difference between the original width and the width after cell migration. All assays were carried out in triplicates independently.

### Chromatin immunoprecipitation

The chromatin immunoprecipitation (ChIP) assay was performed using an EZ-ChIP kit (Millipore, Catalog No. 17–10,461) according to manufacturer’s instructions. The E-cadherin promoter region located −3000 to −1 bp upstream of the transcription start site was amplified. Products were quantified by Real-time PCR method using both the ChIP-enriched DNA and input DNA as template. Enrichment by ChIP was assessed relative to the input DNA and normalized to the level of β-actin. The PCR primers for E-cadherin are listed in Table 1.

### Migration and invasion assay

8-μm pore polycarbonate membrane inserts (Becton Dickinson, Franklin Lakes, NJ, USA) were used to measure the HCC cells’ invasive and migration abilities according to the manufacturer’ s protocol. In short, 2 × 10^6^ cells in 250 μL serum free medium were seeded into the upper chamber and 500 μL medium containing 10% FBS was added to the lower chamber. After 48 h in culture, cells on the upper side were removed by a swab, fixed in 100% methanol for 15 min at room temperature, and then stained by crystal violet. Photographs of five random fields under 200 × magnification were captured for quantification analysis with the double-blind method. Three identical replicates were performed and eventually got a mean values.

### Hematoxylin-eosin stained and immunohistochemistry

Tissues were fixed in 10% neutral formalin and then embedded in paraffin. 4 μm thick sections were prepared by pathological technologist. Hematoxylin-eosin (HE) stain was performed as previous described [27]. For immunohistochemistry (IHC) staining, sections were deparaffinized, rehydrated, and then prepared for antigen retrieval and soaked in 3% H_2_O_2_ for 15 min at room temperature. Subsequently, the above sections were blocked with goat non-specific serum and incubated with GABPA antibody (1:400, 21,542–1-AP, Proteintech) and E-cadherin (1:100, 14472S, CST) at 4 °C overnight and biotin-labeled secondary antibody for 20 min at room temperature. Lastly, the sections were developed by dropwise adding DAB and stained with hematoxylin (Maixin Inc., Fuzhou, China). Evaluation of GABPA and E-cadherin staining in HCC tissue sections was performed refer to the IHC assessment methods used by Motoyuki Hashiguchi et al. previously [28].

### Animal assay

Male nude mice (4 to 5 weeks old) used in our study were purchased from Xiamen University and housed in Xiamen University laboratory animal center under pathogen-free conditions according to the institutional guidelines for animal care. All animal experiments met the National Institutes of Health Guidelines and were approved by the Committee on the Ethics of Animal Experiments of Xiamen University. As previous described [29], mice were randomly assigned into two groups (10 cases for 7402-shCtrl group and 7402-ShGABPA group, respectively). 1.5 × 10^6^ cells were re-suspended in PBS medium and then injected into the subcutaneous of armpit. The mice were sacrificed 40 days later and their lung and liver tissues were collected for metastatic foci examination via pathological stain.

### Statistical analysis

Statistical analyses were performed using SPSS 21.0 (IBM, Chicago, IL, USA) and GraphPad Prism 5.0 (La Jolla, CA, USA) software. The results were expressed as the mean ± SD. Quantitative data were performed by two-related samples Wilcoxon non-parametric test for comparing the difference between two different groups. Categorical data were analyzed by X^2^ Test. Kaplan Meier analysis was used to evaluate the survival difference between subgroups. And the Spearman’s rank correlation analysis was used to examine possible correlations between GABPA and E-cadherin expression. *P* value less than 0.05 was considered as statistical significant.

## Results

### GABPA was downregulated in human HCC tissues and predicted a poor prognosis of HCC patients

**Fig. 1:**
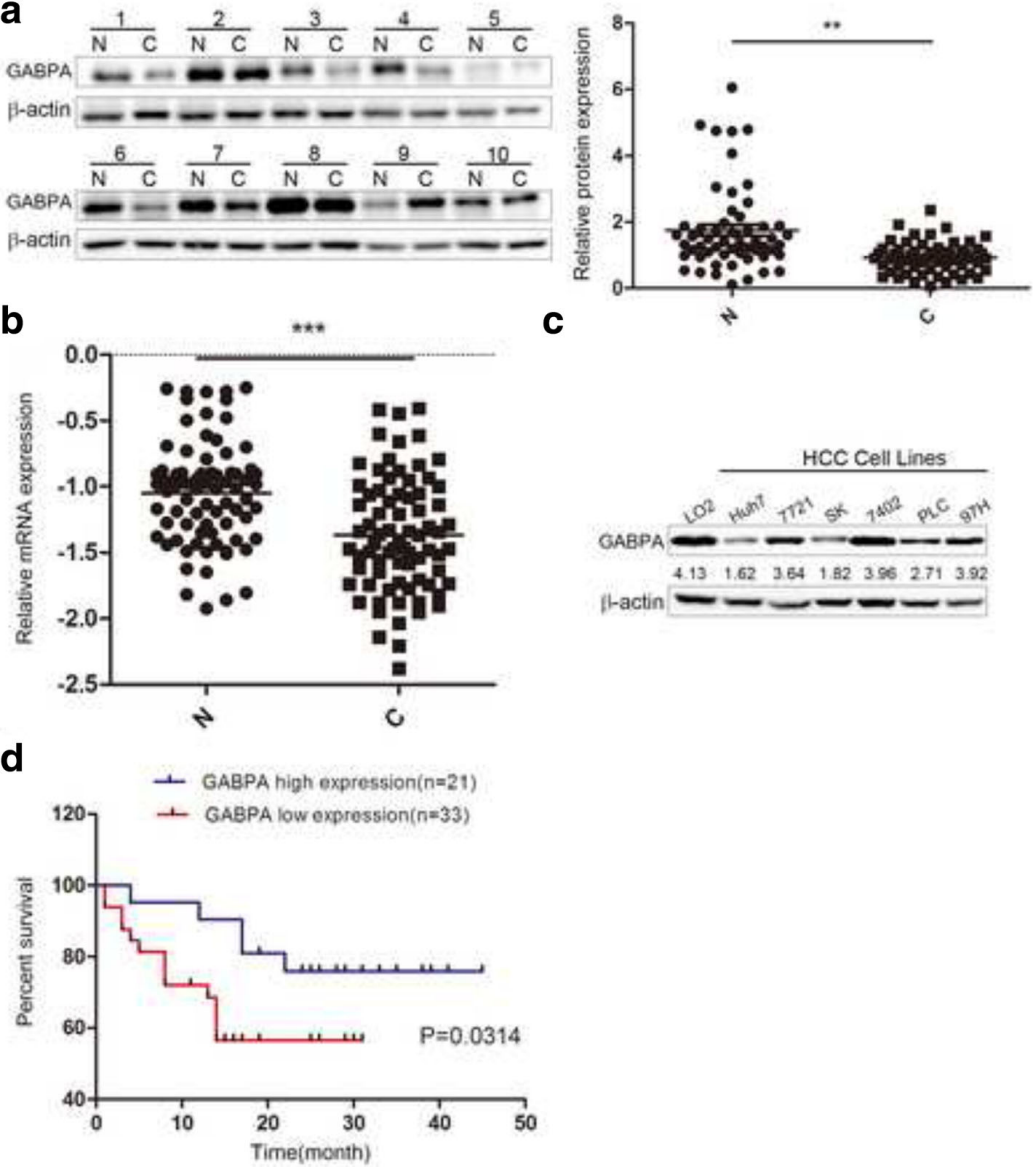
Detection of GABPA expression patterns and clinicopathological significance in HCC cell lines and tissues. **a** Western blot analysis was performed to assess GABPA protein levels in 10 representative HCC tissues (**c**) and paired normal adjacent tissues (N) (*n* = 50). **b** GABPA mRNA expression levels were detected in clinical paired samples by real-time PCR (*n* = 71). **c** GABPA expression levels were consistently decreased in HCC cell lines. **d** Effect of GABPA expression on overall survival by Kaplan-Meier analysis in 54 patients with HCC. (**P < 0.01; ***P < 0.001)

To explore the potential involvement of GABPA in HCC progression, the expression levels of GABPA were measured by western blotting in 50 paired HCC tissues and adjacent noncancerous liver tissues. GABPA was downregulated in HCC specimens compared with its expression in normal tissues (Fig. 1a and Additional file 1: Figure S1). Consistent with this finding, real-time PCR analysis in 71 paired samples showed that GABPA mRNA expression was significantly lower in HCC than in adjacent normal tissues (Fig. 1b). Next, GABPA protein expression was examined in a panel of six widely used human HCC cell lines in comparison to that in the non-malignant cell line LO2. GABPA expression levels were consistently decreased in HCC cell lines (Fig. 1c). The downregulation of GABPA in human HCC tissues and cell lines suggested that GABPA functions as a tumor-suppressor in HCC.

The above findings suggested that GABPA plays a critical role in HCC; therefore, its involvement in HCC was explored further. Firstly, the correlation between GABPA mRNA expression levels and the clinical characteristics of patients was analyzed to evaluate the potential clinical significance of GABPA in HCC patients. The results of the chi-square test indicated that abnormal expression of GABPA in HCC tissues was related to alpha-fetoprotein (AFP) levels (*P* = 0.001), tumor grade (*P* = 0.017), and tumor distant metastasis (*P* = 0.021) (Table 2). Secondly, HCC patients were followed-up and the 54 patients were divided into two groups according to the mRNA expression levels of GABPA as follows: high-GABPA (*n* = 21, with higher GABPA mRNA level compared with paired non-tumor) and low-GABPA (*n* = 33). Kaplan-Meier survival analysis showed that patients with low GABPA expression levels had significantly shorter survival times than those with high GABPA expression (Fig. 1d, *P* = 0.0314).

**Table 2:** Correlation of GABPA mRNA expression with clinic-pathological features in hepatocellular carcinoma

Variables	Category	GABPA (N > C)	Number of case	*P* Value
Age	<50	20 (76.9%)	26	0.356
	≥50	30 (66.7%)	45	
Gender	Male	41 (70.7%)	58	0.917
	Female	9 (69.2%)	13	
Tumor size	<5 cm	18 (69.2%)	26	0.867
	≥5 cm	32 (71.1%)	45	
AFP(ng/ml)	<400	11 (45.8%)	24	0.001*
	≥400	39 (83.0%)	47	
HBsAg	Negative	12 (70.6%)	17	0.986
	Positive	38 (70.4%)	54	
Cirrhosis	Absent	22 (73.3%)	30	0.646
	Present	28 (68.3%)	41	
Tumor grade	Low	45 (76.3%)	59	0.017*
	High	5 (41.7%)	12	
Metastasis	Yes	36 (80.0%)	45	0.021*
	No	14 (53.8%)	26	

*Represent statistical significant

### Knockdown of GABPA promoted HCC cell invasion and migration in vitro, whereas ectopic expression of GABPA had the opposite effect

**Fig. 2:**
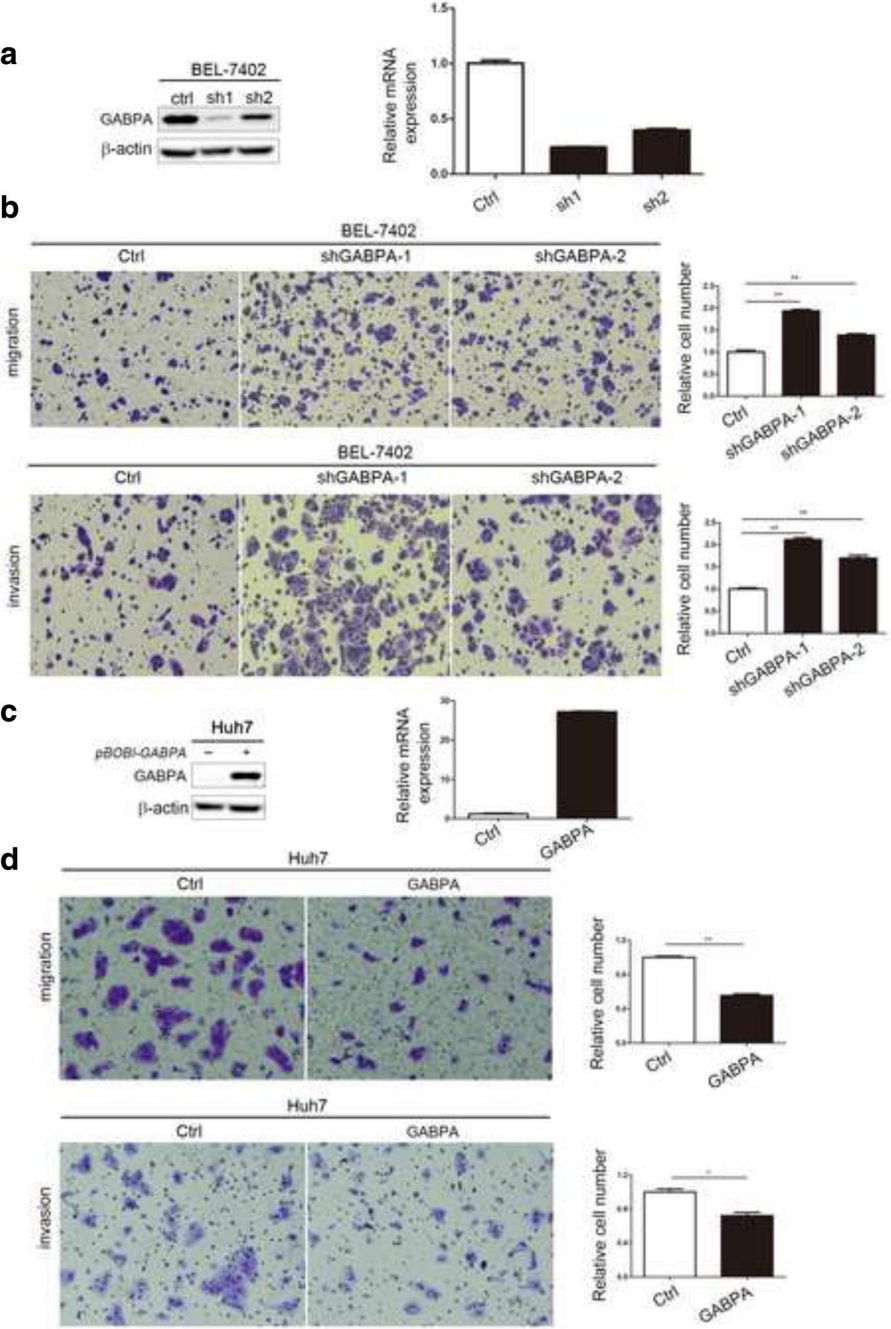
GABPA expression was negatively associated with HCC cell invasion and migration. **a** shRNA-induced GABPA silencing in BEL-7402 cells. β-actin was used as a loading control. **b** Downregulation of GABPA promoted HCC cell migration and invasion. **c** Overexpression of GABPA in Huh7 cells. **d** Ectopic expression of GABPA repressed HCC cell invasion and migration. (*P < 0.05; **P < 0.01)

As poor prognosis of HCC patients is mainly related to tumor migration, we further examined the effect of GABPA on the invasive properties of HCC cells. To address this issue, endogenous GABPA was stably knocked down using a lentivirus vector-based shRNA approach in BEL-7402 cells. The protein and mRNA levels of GABPA were dramatically downregulated in BEL-7402-ShGABPA cells compared with those in the control (Fig. 2a). Migration and invasion chamber assays showed that silencing GABPA dramatically promoted the migratory and invasive capacities of BEL-7402 cells (Fig. 2b), whereas stable overexpression of GABPA by lentiviral vector-mediated transfection in Huh-7 cell lines had the opposite effects on invasion and migration (Fig. 2c and d). Similar results were obtained in SMMC-7721 and SK-Hep1 cells (Additional file 2: Figure S2). Collectively, the above data indicated that GABPA expression was negatively associated with HCC cell invasion and metastatic ability in vitro.

### GABPA regulated E-cadherin protein expression in HCC

**Fig. 3:**
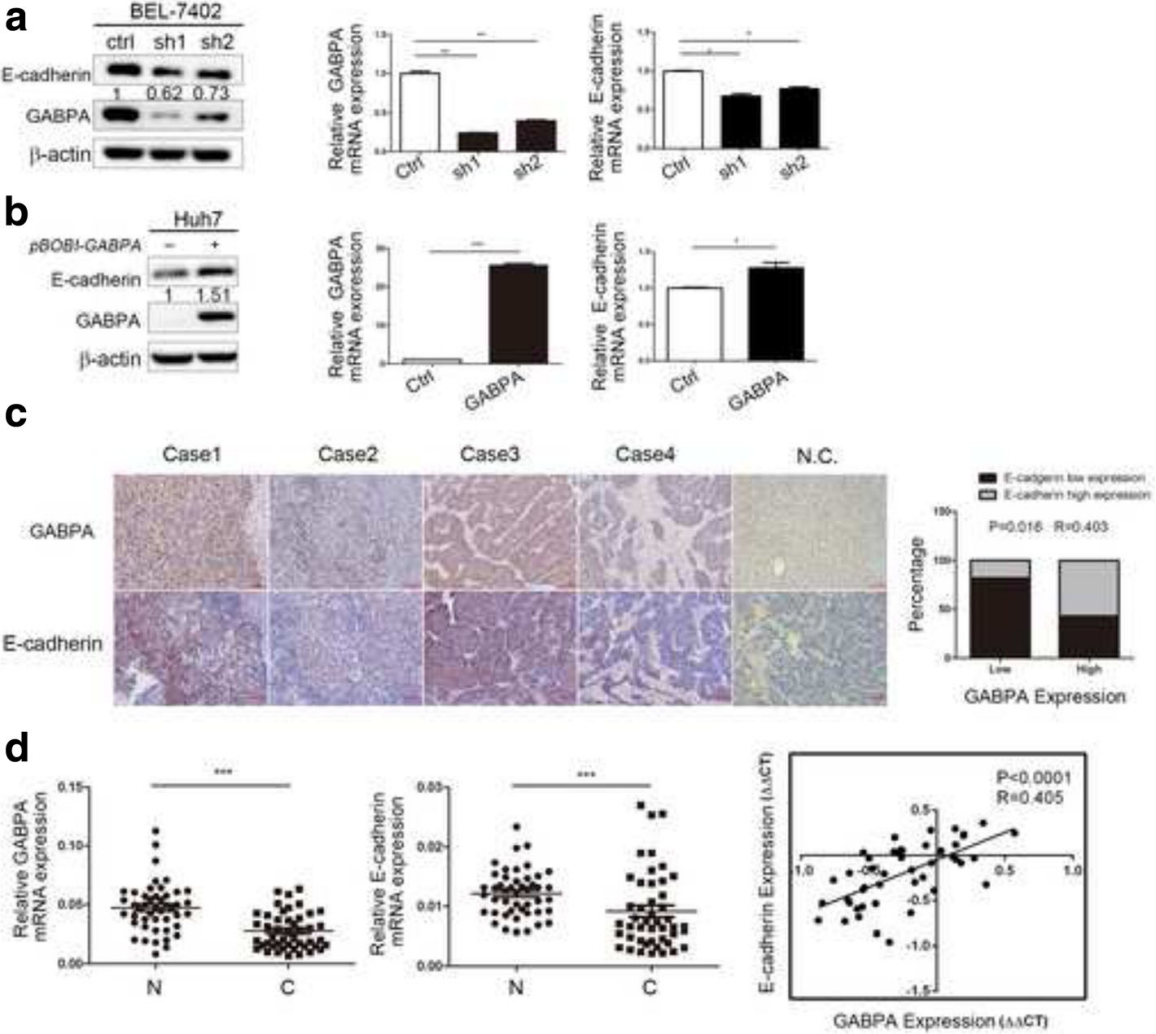
GABPA positively regulated E-cadherin expression. **a** The protein and mRNA level of E-cadherin were significantly downregulated in BEL-7402-ShGABPA compared with BEL-7402-ShCtrl. **b** E-cadherin protein and mRNA levels were increased moderately when GABPA was overexpressed in Huh7 cells. **c** Immunohistochemical staining of GABPA and E-cadherin in four representative HCC clinical tissues. Spearman correlation analysis showed that GABPA expression was positively correlated with E-cadherin. Negative controls were prepared using non-immune rabbit IgG at the same dilution as the primary antibody in normal and tumor samples. **d** GABPA mRNA level in HCC tissues was positively associated with E-cadherin. (*P < 0.05; **P < 0.01)

In the present study, GABPA was negatively correlated with HCC cell migration and invasion potency. However, the underlying molecular mechanism remains unclear. On the basis of the biological function of E-cadherin in HCC tumor metastasis [30], we examined the expression of E-cadherin in GABPA knockdown cell lines by western blotting and real-time PCR. The results showed that E-cadherin was downregulated at the protein and mRNA levels in BEL-7402-ShGABPA compared with BEL-7402-ShCtrl (Fig. 3a). Conversely, ectopic expression of GABPA in Huh7 cells upregulated E-cadherin protein and mRNA expression (Fig. 3b).

Next, the correlation between GABPA and E-cadherin was assessed by immunohistochemistry in 36 HCC tissue samples. Consistent with the results obtained in cell lines, a significant positive correlation between GABPA and E-cadherin expression was detected in HCC tissues (*P* = 0.016, *R* = 0.403, Fig. 3c). Real-time PCR detection of mRNA expression patterns showed similar results, with a positive association between GABPA and E-cadherin at the mRNA level (*P* < 0.0001, *R* = 0.405, Fig. 3d).

### GABPA induced HCC cell migration partly by modulating E-cadherin

**Fig. 4:**
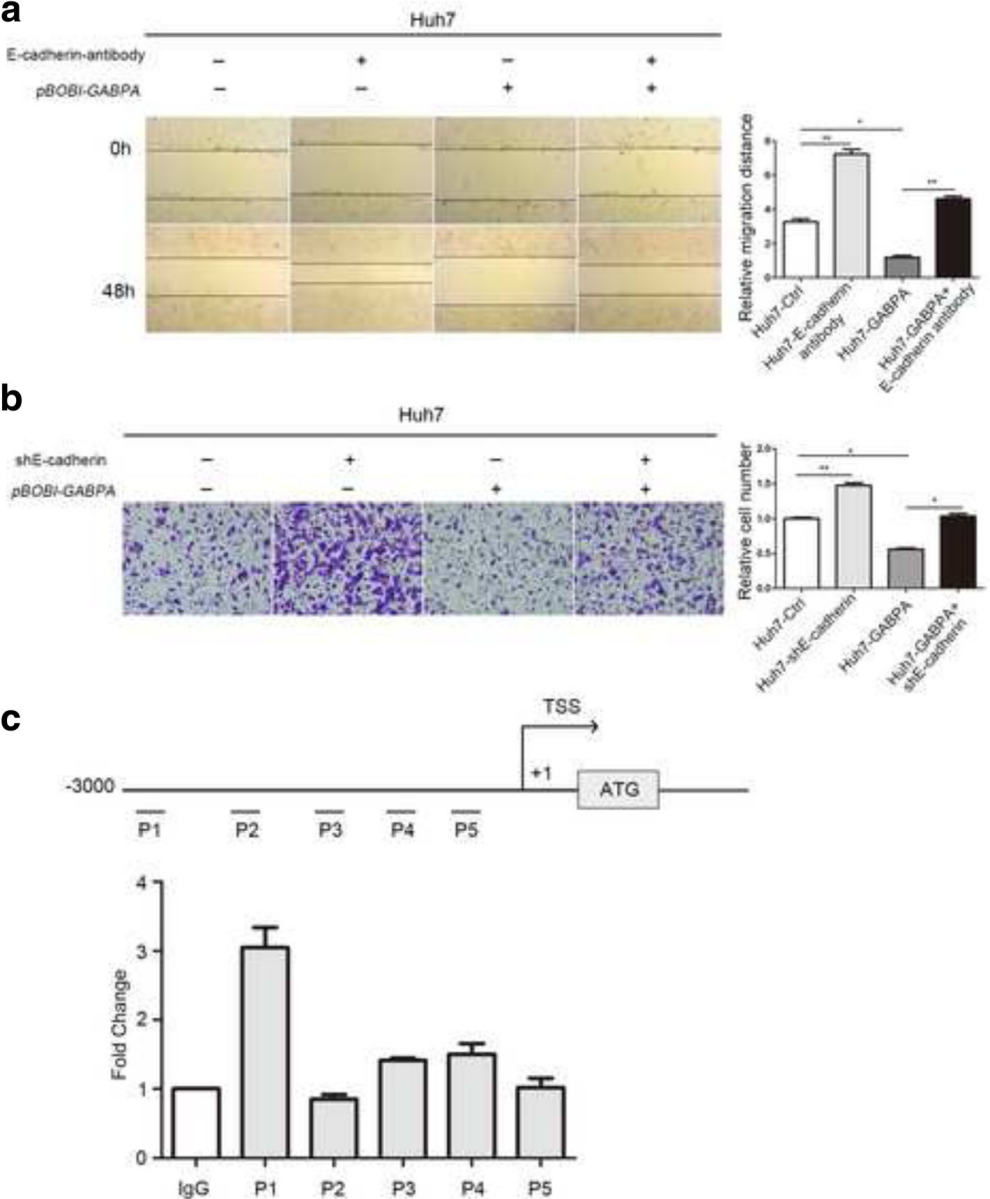
GABPA induced HCC cell migration partly by modulating E-cadherin. **a** GABPA overexpression reduced migration potency (lane 1 vs. 3), whereas addition of E-cadherin antibody to block E-cadherin function partially restored HCC cell migration capacity (lane 3 vs. 4). Lane 2 was used as a positive control. **b** Migration assays showed that GABPA overexpression-induced reduction of migration potency was partially impaired by knockdown of E-cadherin expression. **c** GABPA did not bind directly to the E-cadherin promoter (*P < 0.05, **P < 0.01)

E-cadherin negatively regulates cancer cell invasion and migration. Our results showing that GABPA inhibited HCC cell invasion and modulated E-cadherin expression led us to speculate that GABPA exerts its function in HCC cells by modulating E-cadherin expression. To test this hypothesis, wound healing and restoration of function experiments were performed in four groups of cells: Huh7-Ctrl, Huh7-E-cadherin antibody, Huh7-GABPA, and Huh7-GABPA + E-cadherin antibody. As shown in Fig. 4a, treatment with an E-cadherin specific antibody at 10 μg/mL to block E-cadherin function impaired the effect of GABPA on HCC cell migration. Similarly, knockdown of E-cadherin in Huh7-GABPA + shE-cadherin cells restored the effect of GABPA overexpression on migration potency (Fig. 4b). Taken together, these results confirmed that GABPA repressed HCC cell migration at least partially by regulating E-cadherin.

GABPA is located in the nucleus and binds to multiple gene promoters. Since GABPA positively regulated E-Cadherin mRNA level in our present study, it is possible that GABPA binds to the promoter of E-cadherin and regulates its expression. To test this hypothesis, binding of the endogenous GABPA protein to the E-cadherin promoter was analyzed by ChIP assays in vitro. However, the primer set did not induce a strong DNA amplification of the E-cadherin promoter region (Fig. 4c). Collectively, these results indicated that GABPA does not directly bind to the E-cadherin promoter.

### Knockdown of GABPA promoted HCC metastasis in vivo

**Fig. 5:**
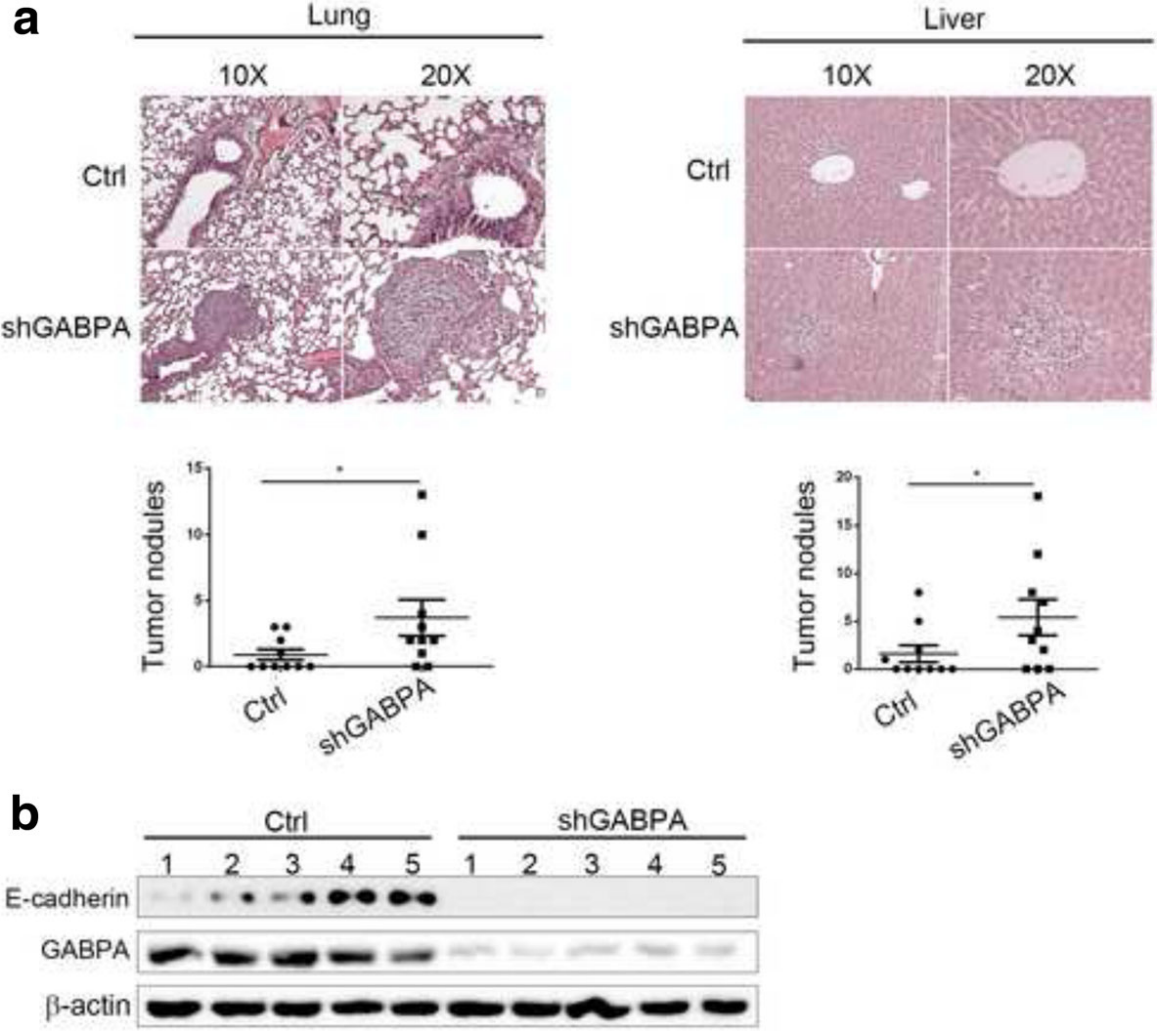
GABPA knockdown enhanced HCC metastasis in vivo. **a** Mice injected with 7402-shCtrl cells had fewer pulmonary and intra-hepatic micro-metastatic nodules than those injected with 7402-shGABPA. **b** Western blot analysis of the expression of GABPA and E-cadherin protein levels in 7402-shGABPA and 7402-shCtrl tumors. (*P < 0.05)

Next, the effects of GABPA downregulation were examined in vivo in a xenograft model. 7402-shGABPA and 7402-shCtrl cells were injected into nude mice. At 40 days after implantation, mice were sacrificed and the metastatic nodules were counted in a double-blind manner. As shown in Fig. 5a, injection of 7402-shGABPA cells increased the number of metastatic tumors in the lungs and liver of mice, and these results were confirmed by histology and HE staining. However, there was no significant difference in the tumor size between the groups (Additional file 3: Figure S3a). Injection of mice with SK-Hep1-Ctrl and SK-Hep1-GABPA overexpressing cells showed the opposite results (Additional file 3: Figure S3b). To correlate the biological response with the mechanisms identified in HCC cells, E-cadherin protein levels were assessed by western blotting. As shown in Fig. 5b, knockdown of GABPA significantly downregulated E-cadherin in transplanted tumor tissues. The in vivo results supported the hypothesis that GABPA plays a critical role in suppressing HCC cell migration and invasion.

## Discussion

HCC is a common malignancy and its incidence and mortality are increasing worldwide. Despite advances in the surgical and medical treatment of HCC and extensive research into the mechanisms underlying HCC metastasis, the mortality from HCC remains high and the prognosis of patients is poor [31]. Tumor invasion, metastatic dissemination, and recurrence are the major causes of the poor clinical outcome of HCC patients [32–34]. Therefore, it is vital to identify metastasis-associated biomarkers and elucidate the mechanisms underlying HCC metastasis to develop effective therapeutic strategies to improve the quality of life of HCC patients.

Studies indicate that GABPA is involved in embryonic development, innate and acquired immunity, myeloid and hematopoietic stem cell differentiation, and cell cycle progression among other functions. However, to the best of our knowledge, the regulatory roles and mechanisms of GABPA in HCC cell migration and its clinical pathological significance have not been reported to date.

In the present study, we used a series of techniques to validate the role of GABPA in HCC metastasis and examined the potential underlying mechanisms. We demonstrated for the first time that GABPA is negatively associated with HCC progression, as indicated by the following results: First, GABPA protein and mRNA were downregulated in human HCC tissues compared with adjacent noncancerous tissues. GABPA was also consistently downregulated in HCC cell lines. Second, in vitro experiments showed that shGABPA vector-mediated GABPA knockdown in HCC cell lines markedly promoted cell migration and invasion, whereas ectopic expression of GABPA had the opposite effects. Third, in vivo assays in a xenograft model confirmed these results, as the number of metastatic tumors in the lungs and liver was higher in the sh-GABPA group than in the control group. Consistent with the tumor suppressive role of GABPA in cells, GABPA downregulation was associated with aggressive clinicopathological characteristics in HCC patients. Moreover, a low GABPA mRNA level was significantly associated with decreased survival time and worse prognosis in HCC patients.

A previous study indicated that GABPA exerts paradoxical roles in regulating cell migration and invasion. GABPA inhibits the migratory properties of vascular smooth muscle cells by controlling the expression of the kinase KIS [18]. Odrowaz and Sharrocks reported that GABPA plays a complex role in controlling breast epithelial cell migration by directly affecting the expression of the RAC2 and KIF20A genes [24]. Therefore, the effect of GABPA on cancer cell migration and invasion may be cancer and tissue specific and may involve different signaling pathways in different cells.

In the present study, we investigated the potential mechanisms underlying the effect of GABPA on HCC cell migration and invasion. Previous studies showed that EMT plays a pivotal role in HCC tumor metastasis [35, 36] and contributes to early stage dissemination of cancer cells [37]. We therefore examined the effect of GABPA on EMT markers and showed that GABPA knockdown downregulated E-cadherin at the protein and mRNA levels. However, overexpression of GABPA had a moderate effect on the mRNA and protein expression of E-cadherin. We speculated that shRNA-mediated GABPA knockdown led to a lower amount of GABPB indirectly binding to the E-cadherin promoter and inactivating its transcription, whereas overexpression of GABPA could not enhance the transcription of the E-cadherin gene significantly because of the limitation of the amount of GABPB, which forms a functional GABP transcription factor complex with GABPA. However, future studies are needed to verify these hypotheses.

GABPA was previously shown to physically interact with methyltransferase like 23 to regulate thrombopoietin and ATP5B gene expression [38]. Additionally, Lucas et al. showed that GABPA was selectively enriched at HS2 in human cells, and its occupancy was inversely correlated with CpG island methylation of the TMS1 gene [39]. These results suggest the epigenetic regulation of GABPA expression. In the present study, we accidentally found that EZH2 was negatively regulated by GABPA (Additional file 4: Figure S4a). Han et al. reported that EZH2 suppresses E-cadherin expression and promotes pancreatic cancer cell migration [40]. Therefore, we speculated that GABPA may indirectly promote E-cadherin expression because of its effect on EZH2. To test this hypothesis, the expression level of E-cadherin was detected by western blotting in three groups of cells: Huh7-Control, Huh7-GABPA, and Huh7 cells overexpressing GABPA and EZH2. As shown in Fig. S4b, upregulation of GABPA increased E-cadherin protein levels, and this effect was moderately restored by EZH2. These results demonstrate that GABPA regulates E-cadherin via EZH2. However, the specific mechanism needs further clarification.

## Conclusion

In summary, in vitro and in vivo experiments showed that GABPA was downregulated in HCC tissues and cell lines. Low GABPA expression was associated with aggressive clinicopathological characteristics and poor survival in HCC patients. GABPA suppressed the migration and invasion of HCC cell lines by regulating E-cadherin expression, and restoration experiments showed that GABPA positively regulated E-cadherin expression by modulating EZH2. The present study provides evidence supporting a link between the biological activity of GABPA and HCC invasion and migration. Our research indicates that GABPA may serve as a potential marker for HCC and could be useful for the development of effective treatments against HCC.

## Additional files

Additional file 1: Figure S1. **a** a Expression patterns of the GABPA protein in 50 paired clinical samples. (C represents HCC tissues and N represents adjacent noncancerous liver tissues). **b** Differences in the expression of GABPA protein and mRNA in paired clinical samples. **c** Relative expression levels of GABPA mRNA in HCC cell lines. (TIFF 19116 kb)

Additional file 2: Figure S2. **a** and **b** Knockdown of GABPA in SMMC-7721 promoted cell invasion and migration. **c** and **d** Ectopic expression of GABPA in SK-Hep1 inhibited invasion and migration of HCC cells. (*P < 0.05, **P < 0.01). (TIFF 25518 kb)

Additional file 3: Figure S3. **a** There was no significant difference in tumor size between 7402-shGABPA and 7402-shCtrl groups. **b** Injection with SK-Hep1 GABPA-overexpressing cells dramatically decreased the number of metastatic tumors in the lungs and liver compared with those in the control. (*P < 0.05). (TIFF 13578 kb)

Additional file 4: Figure S3. **a** Downregulation of GABPA upregulated EZH2 at the protein level, whereas overexpression of GABPA had the opposite effect. **b** GABPA overexpression upregulated E-cadherin was partially restored by EZH2. (TIFF 7088 kb)

## Abbreviations

cDNA: complementary DNA
ChIP: Chromatin immunoprecipitation
CML: chronic myelogenous leukemia
EMT: Epithelial mesenchymal transition
ETS: E twenty six
FBS: Fetal bovine serum
HCC: Hepatocellular carcinoma
HE: Hematoxylin-eosin
IHC: Immunohistochemistry
MEFs: Mouse embryonic fibroblasts
mRNA: Messenger RNA
PBS: Phosphatebuffered saline
ShRNA: Short-hairpin RNA
