# Supplementary figures and images for: GABPA predicts prognosis and inhibits metastasis of hepatocellular carcinoma
Source: BMC Cancer. 2017 May 26;17:380. doi: 10.1186/s12885-017-3373-7 (PMC5446731; doi:10.1186/s12885-017-3373-7)

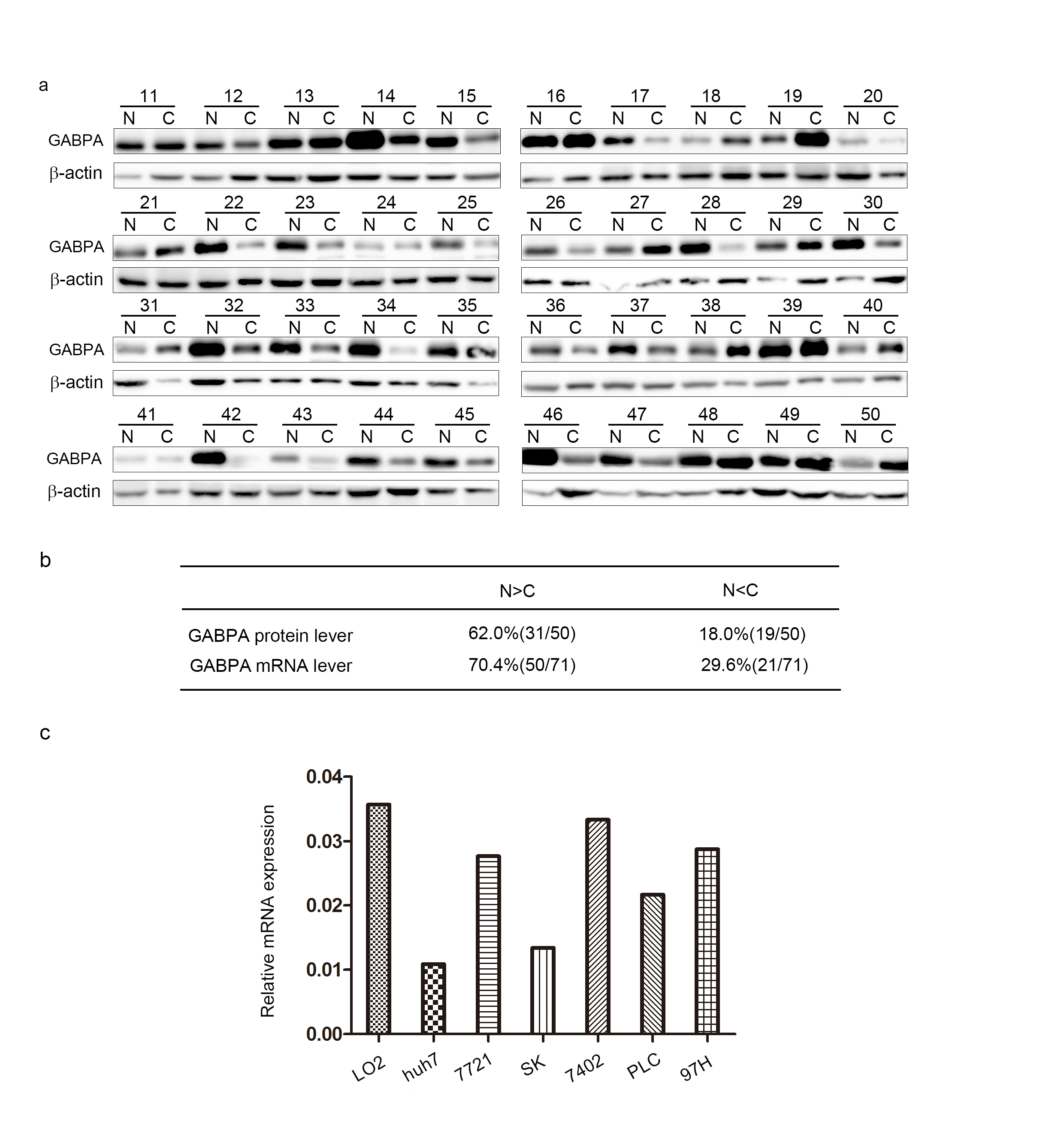

Supplement: Supplementary file 1 — a a Expression patterns of the GABPA protein in 50 paired clinical samples. (C represents HCC tissues and N represents adjacent noncancerous liver tissues). b Differences in the expression of GABPA protein and mRNA in paired clinical samples. c Relative expression levels of GABPA mRNA in HCC cell lines. (TIFF 19116 kb) [file 12885_2017_3373_MOESM1_ESM.tif]

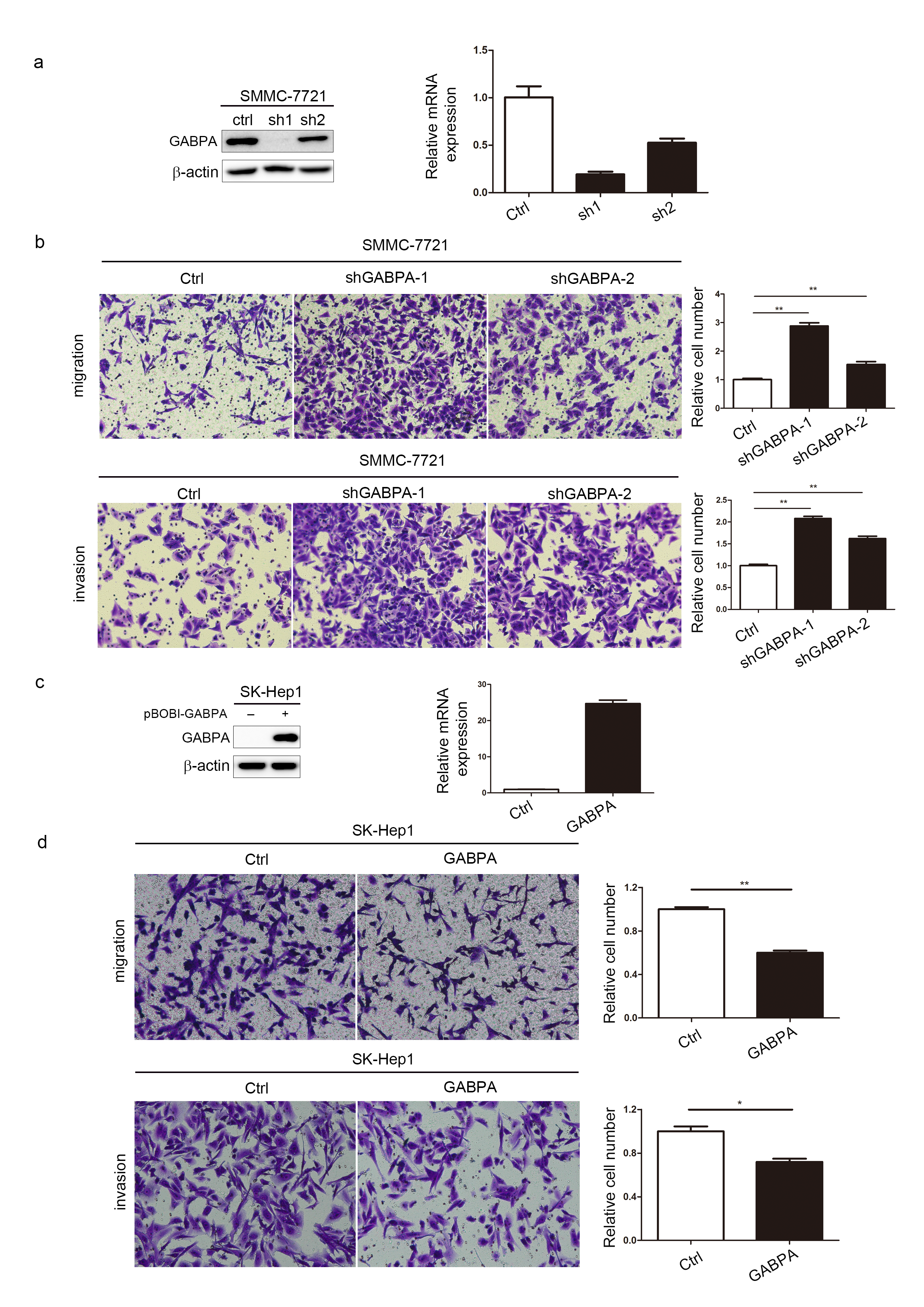

Supplement: Supplementary file 2 — a and b Knockdown of GABPA in SMMC-7721 promoted cell invasion and migration. c and d Ectopic expression of GABPA in SK-Hep1 inhibited invasion and migration of HCC cells. (*P < 0.05, **P < 0.01). (TIFF 25518 kb) [file 12885_2017_3373_MOESM2_ESM.tif]

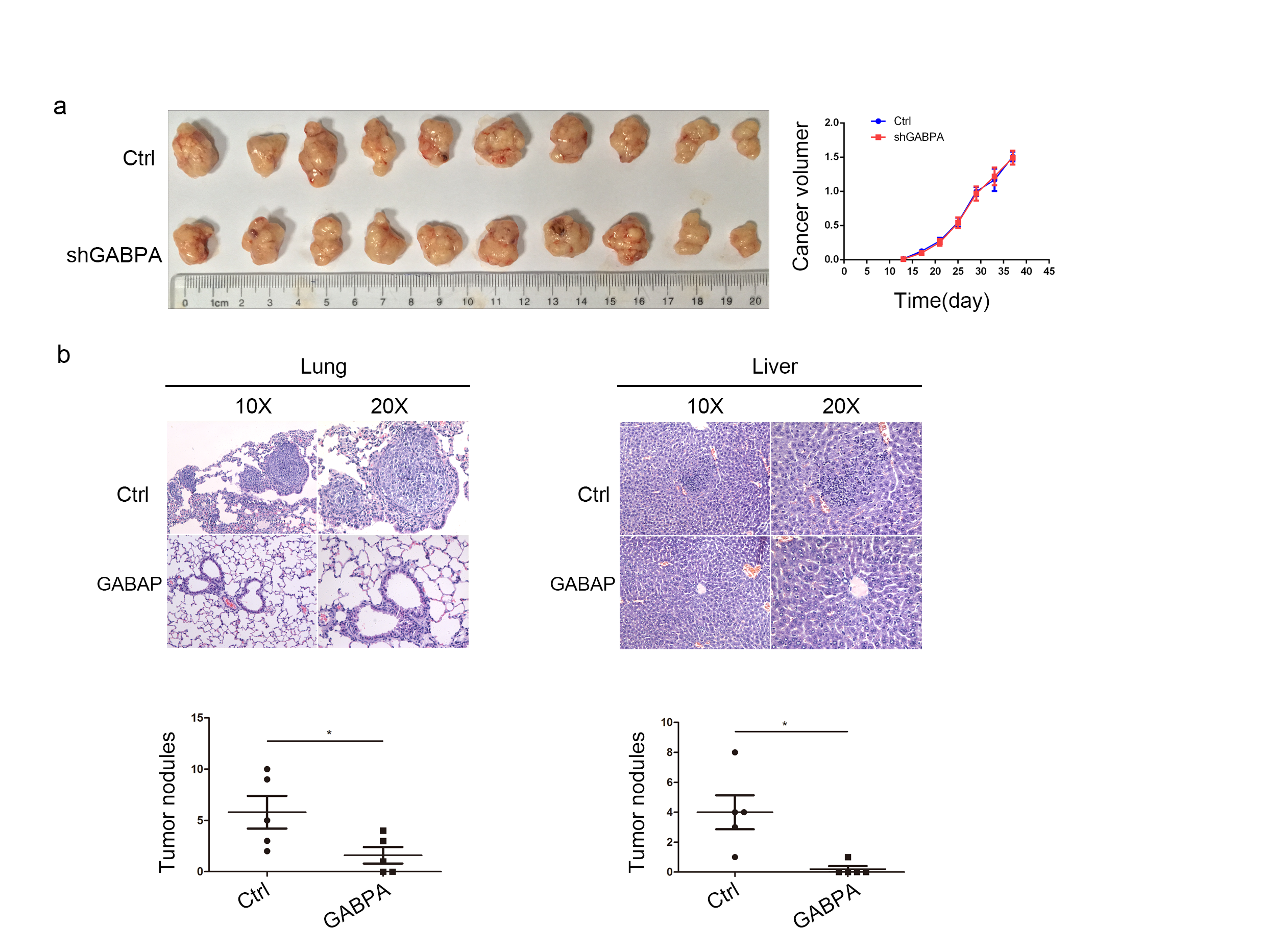

Supplement: Supplementary file 3 — a There was no significant difference in tumor size between 7402-shGABPA and 7402-shCtrl groups. b Injection with SK-Hep1 GABPA-overexpressing cells dramatically decreased the number of metastatic tumors in the lungs and liver compared with those in the control. (*P < 0.05). (TIFF 13578 kb) [file 12885_2017_3373_MOESM3_ESM.tif]

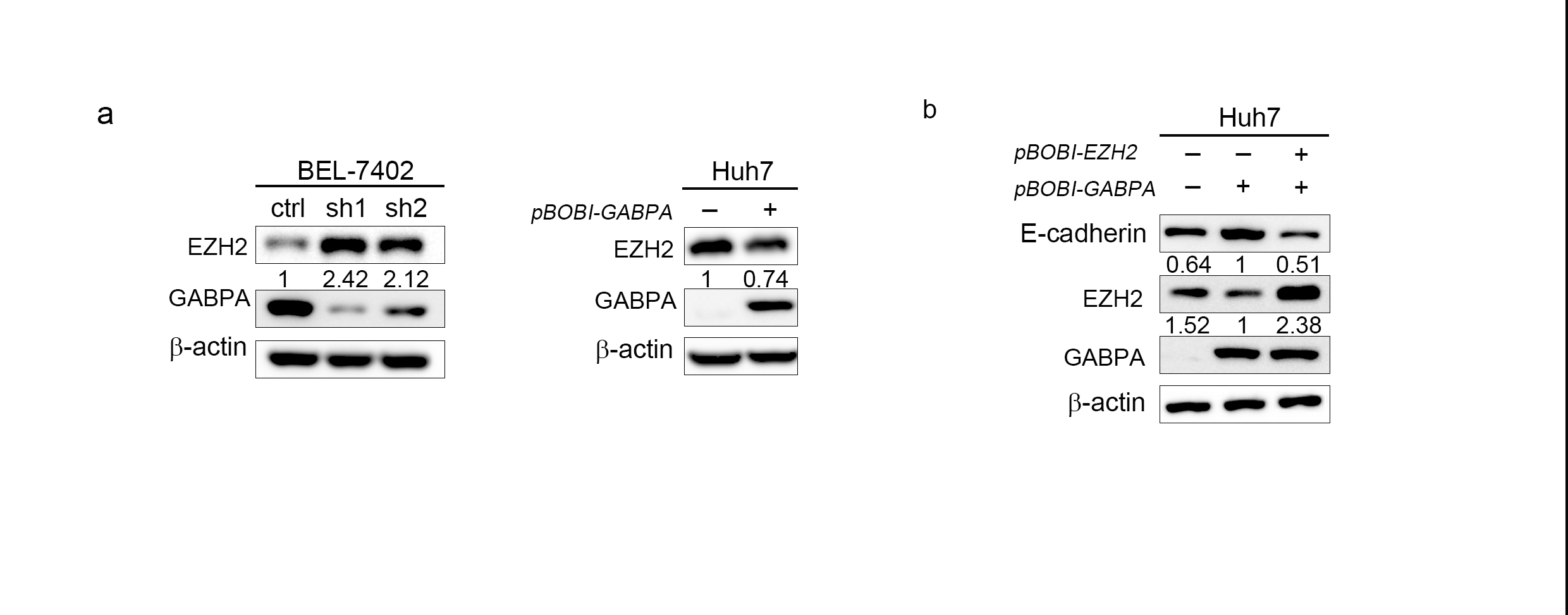

Supplement: Supplementary file 4 — a Downregulation of GABPA upregulated EZH2 at the protein level, whereas overexpression of GABPA had the opposite effect. b GABPA overexpression upregulated E-cadherin was partially restored by EZH2. (TIFF 7088 kb) [file 12885_2017_3373_MOESM4_ESM.tif]
